# Surface stratification determines the interfacial water structure of simple electrolyte solutions

**DOI:** 10.1038/s41557-023-01416-6

**Published:** 2024-01-15

**Authors:** Yair Litman, Kuo-Yang Chiang, Takakazu Seki, Yuki Nagata, Mischa Bonn

**Affiliations:** 1 Max Planck Institute for Polymer Research, Mainz, Germany; 2 Yusuf Hamied Department of Chemistry, University of Cambridge, Cambridge, UK

**Keywords:** Surface spectroscopy, Molecular dynamics

## Abstract

The distribution of ions at the air/water interface plays a decisive role in many natural processes. Several studies have reported that larger ions tend to be surface-active, implying ions are located on top of the water surface, thereby inducing electric fields that determine the interfacial water structure. Here we challenge this view by combining surface-specific heterodyne-detected vibrational sum-frequency generation with neural network-assisted ab initio molecular dynamics simulations. Our results show that ions in typical electrolyte solutions are, in fact, located in a subsurface region, leading to a stratification of such interfaces into two distinctive water layers. The outermost surface is ion-depleted, and the subsurface layer is ion-enriched. This surface stratification is a key element in explaining the ion-induced water reorganization at the outermost air/water interface.

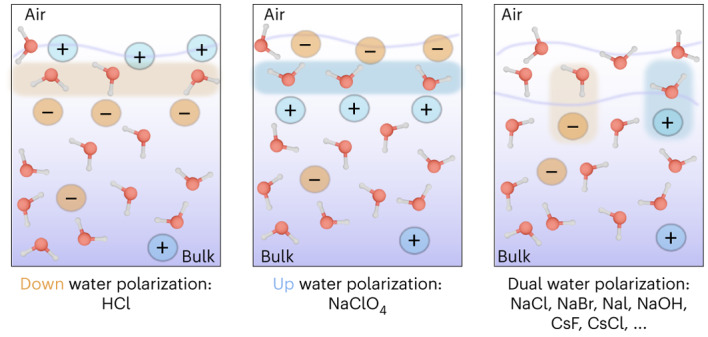

## Abstract (web summary)

The organization of electrolytes at the air/water interface affects the structure of interfacial water and therefore numerous natural processes. It has now been demonstrated that the surface of an electrolyte solution is stratified and consists of an ion-depleted outer surface and an ion-enriched subsurface layer, jointly determining the water interfacial structure.

## Main

Around 70% of the Earth’s surface is covered by ocean water. The evaporation and heterogeneous aerosol formation of such electrolyte-rich solutions play an important role in atmospheric chemistry and climate science^1–3^. The physicochemical processes occurring on the surface of electrolyte solutions are ultimately determined by the molecular structure of such solutions at the air/liquid interfaces. Thus, a microscopic knowledge of the ion distributions and molecular orientation at the arguably simplest air/solution interface is of paramount importance for the development of environmental models^2,4^, and also serves as a starting point for understanding more complex interfaces of liquid solutions in contact with electrodes, membranes or minerals^5–7^.

A widespread molecular picture of the surface of aqueous electrolyte solutions is based on results obtained with surface-specific spectroscopic techniques and force-field-based molecular dynamics (MD) simulations^8–11^. In brief, polarizable heavy ions, such as Br^−^ and I^−^, accumulate at the interfacial region, while less polarizable ions, such as F^−^ and Na^+^, are depleted from the interface and remain buried in the solution. This differential distribution of anions and cations is presumed to create the so-called electric double layer (EDL), an ionic double-layer structure or simply a double layer^8,9,12–14^. The assumption of the formation of an EDL at air/liquid interfaces has played a fundamental role in describing aqueous electrolyte surfaces^11,15^.

It is challenging to obtain molecular-level insights into the local ion distribution and water structure at the surface of electrolyte solutions. Vibrational sum-frequency generation (VSFG) is a surface-specific technique that directly probes the response from molecular vibrations interfaces. Atomic ions support no vibrations, so their behaviour can only be inferred indirectly from the VSFG response of the water, typically invoking the EDL picture. VSFG signal intensity variations in the 3,000–3,600 cm^−1^ region upon adding salt have been attributed to excess accumulation of cations or anions at the interface^8,13,14,16–18^. Yet, discrepancies have recently become evident, and a unifying picture is absent in the literature. For example, although most polarizable force field-based calculations predict a strong enhancement of the I^−^ concentration at the interface^19^, in agreement with measured surface excess free energy^20^, measurements reported by Raymond and colleagues^21^ concluded a lack of detectable surface enhancement of polarizable anions through a careful design of isotopic dilution experiments, in agreement with more recent ab initio simulations^22^.

Progress in this area is restricted by several challenges: (1) low SFG signal levels from these types of interface; (2) the necessity for determining the signal phase (by heterodyne detection) to record unambiguous data; (3) the insufficiency of experimental data alone to disentangle spectral components in aqueous solutions unambiguously, as the water VSFG response is often broad and featureless^23^.

In this Article we overcome these challenges by combining high-level heterodyne-detected VSFG (HD-VSFG) data with neural network (NN)-aided ab initio MD (AIMD) simulations and, to obtain a unifying picture, we study the structure of the interface of ten electrolyte solutions, namely HCl, NaOH, CsF, NaF, NaCl, NaBr, NaI, MgCl_2_, Na_2_SO_4_, MgSO_4_ and NaClO_4_. HD-VSFG gives direct access to Im(*χ*^(2)^) and allows disentangling the non-resonant background in the HD-VSFG spectra, thereby revealing subtle but essential details in the actual resonant spectra. By systematically changing the salt concentration and comparing HD-VSFG spectra with simulated spectra, we show that the formation of an EDL cannot account for the HD-VSFG spectra of the sodium halide salts and other electrolytes, the only exceptions among the considered electrolytes being HCl and NaClO_4_, for which the hydrated proton and the perchlorate have very high surface propensity. Combined experimental data and simulations with ab initio quality prove that subsurface enrichment of ions a few ångstroms below the air/electrolyte solution dictates the interfacial aqueous structure. This depiction provides a more accurate understanding of the interfacial structure of most typical electrolyte solutions, highlighting that the conventional classifications of ions’ surface propensity as ‘surface enriched’ or ‘surface depleted’ are only two limiting scenarios within a broader range of behaviours.

## Results and discussion

### Shortcomings of the EDL model for air/electrolyte interfaces

**Fig. 1:**
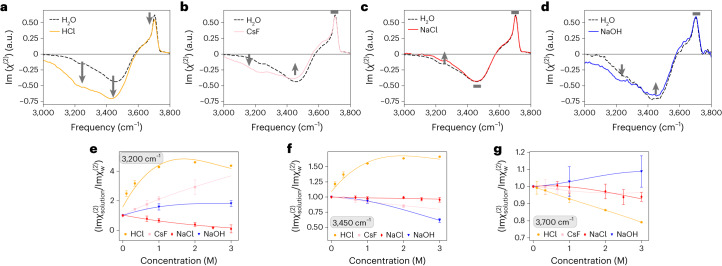
Experimental HD-VSFG spectra at the air/electrolyte solutions interface. **a**–**d**, HD-VSFG spectra for 1 M HCl solution (orange; **a**), 1.5 M CsF solution (pink; **b**), 1.5 M NaCl solution (red; **c**) and 1.0 M NaOH solution (blue; **d**). Data are presented as mean values over three (HCl), three (CsF), four (NaCl) and nine (NaOH) independent datasets. Error bars represent the corresponding 95% confidence interval assuming a two-sided Student’s distribution. The free and hydrogen-bond O–H bands are altered with respect to the pure water reference for the HCl case (**a**), whereas for the CsF and NaCl cases, only the hydrogen-bonded bands are modified. In all panels, the water VSFG spectra of water (black) are shown for comparison. The direction of the spectral changes is highlighted with arrows, and grey rectangles depict negligible spectral changes. **e**–**g**, Concentration dependence of the HD-VSFG amplitudes, expressed as the ratio of electrolyte solution and water signals (${\rm{Im}}{\chi }_{{\rm{solution}}}^{(2)}/{\rm{Im}}{\chi }_{{\rm{w}}}^{(2)}$), at 3,200 (**e**), 3,450 (**f**) and 3,700 cm^−1^ (**g**), showing that the water structure at these air/electrolyte interfaces is qualitatively different. Lines represent a polynomial fit used to guide the eye without physical meaning. Source data

Figure 1 shows the Im(*χ*^(2)^) spectra for water and different electrolytes solutions (Fig. 1a–d). We first focus on the spectrum at the water–air interface. The spectrum of pure water shows a sharp positive peak centred at 3,700 cm^−1^ and a negative signal around 3,200–3,500 cm^−1^. The 3,700 cm^−1^ peak arises from the free O–H group of the topmost interfacial water, and the 3,200–3,550 cm^−1^ band arises from the hydrogen-bonded O–H group of the interfacial water. The positive (negative) sign of the O–H stretch mode indicates that the O–H group points up to the air (down to the bulk).

When compared to the data of pure water, the spectrum of the HCl solution shows a decrease in the free O–H stretch band (less positive) and a decrease of the hydrogen-bonded stretch band (more negative). The decrease of the free O–H peak is an unambiguous indication that protons reside on the topmost layer by capping the free O–H groups with hydronium ions. The surface propensity of protons has been confirmed experimentally^24,25^ and theoretically^26^ in the past. In contrast to the HCl solution case, the Im(*χ*^(2)^) spectra for the NaCl, NaOH and CsF solutions show that these ions modify the hydrogen-bonded band by enhancing and/or reducing their amplitudes, but the free O–H band remains largely unaffected. The unchanged free O–H peak manifests that the free O–H groups of the interfacial water molecules are not capped by these ions.

Figure 1e–g shows the concentration dependence of the 3,200 cm^−1^, 3,450 cm^−1^ and 3,700 cm^−1^ amplitudes (the free O–H peak, and the high- and low-frequency sides of the hydrogen-bonded O–H stretch bands, respectively). Cl^−^, OH^−^ and F^−^ ions do not affect the free O–H peak amplitude significantly within the considered concentration range, unlike the hydronium ions. In contrast, the hydrogen-bonded O–H band amplitudes change significantly for all the ions, although with different magnitudes and signs. These results question the use of one unique model to describe the diverse behaviour of ions at the interface.

Note that our Im(*χ*^(2)^) spectra of the NaCl and NaOH solutions, as well as the pure water, differ from the reported data, particularly in the <3,200 cm^−1^ region^16,17,27,28^. Phase inaccuracies in the early measurement created an artificial positive peak below 3,200 cm^−1^. Thanks to the recent procedure of accurate phase determination, we could obtain the phase accurately in our HD-VSFG spectra. Indeed, our air/water interface data agree with recent reports^28,29^.

### The case of the air/NaOH(aq.) interface

To explore why the free O–H peak is unchanged, but the hydrogen-bonded band is changed, upon the addition of salt, we carried out theoretical calculations of the VSFG spectra. Among NaCl, CsF and NaOH, we decided to investigate the NaOH spectrum in more detail by simulating its HD-VSFG spectra, because NaOH serves as a representative of the electrolytes that do not perturb the free O–H band, and it shows the most drastic change of the HD-VSFG features. Calculation of the VSFG spectra for water is at least ten times more expensive than bulk spectroscopies such as Raman or infrared (IR) spectroscopy^30^, because the signal comes from only very few water layers, and the signal arising from bulk should correctly be averaged to zero. Moreover, the description of bond-breaking and -formation events, as well as the need for accurate spectra, demand going beyond classical force fields and performing ab initio calculations^22,23^. The latter demands a considerably greater computational effort, which explains why ab initio calculations of SFG spectra are scarce in the literature. To overcome this daunting computational cost, we carried out NN-AIMD simulations^31,32^, which allowed us to perform the required simulations at a fraction of the computational cost, while preserving the underlying quantum-mechanical accuracy.

**Fig. 2:**
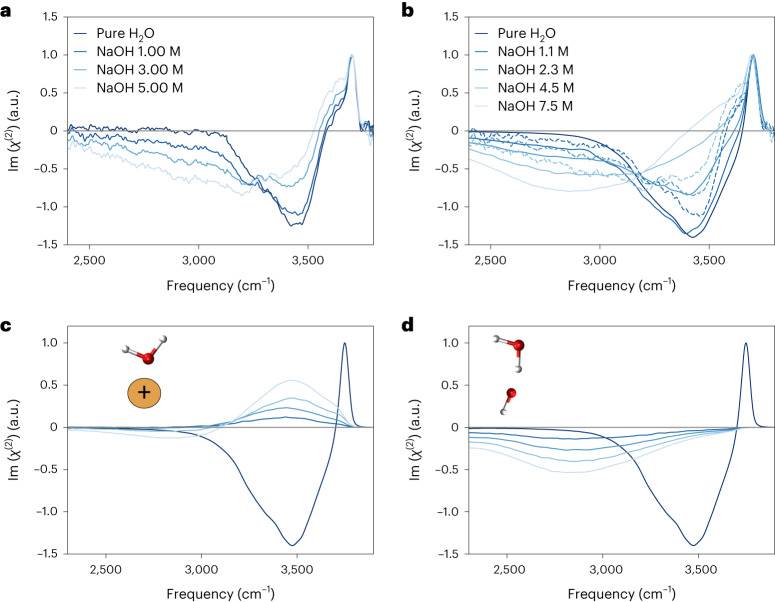
Experimental and theoretical VSFG spectra of NaOH aqueous solutions at room temperature. **a**, Experimental imaginary part of *χ*^(2)^ spectra obtained for water, 1.00 M NaOH, 3.00 M NaOH and 5.00 M NaOH aqueous solutions. **b**, Theoretical prediction based on the ssVVCF methodology^30^. Concentrations are obtained from the bulk region of the slab. Linearly scaled experimental data are depicted with dashed lines to ease comparison. **c**, Contribution to the theoretical spectra by the water molecules in the vicinity of Na^+^ ions. **d**, Contribution to the theoretical spectra by the OH bonds coordinating OH^−^ anions. **c** and **d** disentangle the opposing mechanisms of the spectral changes and allow identification of their molecular origin. The pure water spectrum is shown in all panels in black as a reference. The theoretical spectra have been corrected by the frequency dependence of the transition dipole and polarizability moments^60,61^. A reference zero line has been added in grey. Source data

Figure 2a,b shows the experimental and theoretical HD-VSFG data, respectively. Comparison of experiment and simulation shows that our simulations capture the two main spectral changes correctly. For both sets of spectra, the addition of NaOH ions triggers a decrease in the hydrogen-bonded negative signal between 3,300 cm^−1^ and 3,600 cm^−1^—it becomes less negative—and the emergence of a broad-continuum negative signal in the 2,400–3,200 cm^−1^ region. The free O–H remains unperturbed. The simulations capture the main trends observed experimentally and reproduce the described spectral changes with increasing base concentration. They also lack the shoulder at 3,600 cm^−1^ arising from the asymmetric stretching mode in water molecules, which donate two hydrogen bonds and accept one^33^, due to the well-known limitations of the surface-specific velocity–velocity correlation function (ssVVCF) approximation^30^. Unlike previous simulations based on classical force fields^34^, our NN-AIMD results correctly predict no SFG signal for pure water below 3,000 cm^−1^. Further validation tests are provided in Supplementary Information.

Having validated our theoretical framework, we proceed with an analysis of the simulated spectra. Figure 2c,d present the Im(*χ*^(2)^) spectra contributed by selected water molecules in the proximity of Na^+^ and OH^−^, respectively. Water molecules coordinated to Na^+^ ions contribute to a positive signal between 3,200 and 3,800 cm^−1^, and the O–H bonds of water molecules coordinated to OH^−^ anions are responsible for the negative <3,500 cm^−1^ continuum. The latter assignment is consistent with previous studies of NaOH in bulk^35–37^ and water clusters^38,39^, where the <3,500 cm^−1^ continuum band is also attributed to the solvation of OH^−^ species. Note that the continuum that extends below 2,500 cm^−1^ does not arise from the bulk *χ*^(3)^ contributions, because the bulk *χ*^(3)^ contributions have only a small contribution below 3,000 cm^−1^, unlike the spectra shown in Fig. 2d ref. ^40,41^.

This spectral decomposition strongly indicates that the HD-VSFG spectra at the electrolyte air/solution interface $({\chi }_{{\rm{solution}}}^{(2)})$ can be described as the sum of the VSFG spectrum of (unperturbed) water at the air/water interface (${\chi }_{{\rm{water}}}^{(2)}$), of water oriented by the cation (${\chi }_{\rm{cation}}^{(2)}$), and that of water oriented by the anion (${\chi }_{\rm{anion}}^{(2)}$):

1 $${\chi }_{{\rm{solution}}}^{(2)}={\chi }_{{\rm{water}}}^{(2)}+c{\chi }_{{\rm{electrolyte}}}^{(2)}{=\chi }_{{\rm{water}}}^{(2)}+c\left({\chi }_{\rm{cation}}^{(2)}+{\chi }_{\rm{anion}}^{(2)}\right)$$

where *c* is a parameter proportional to the salt concentration, and we have defined the electrolyte contribution, ${\chi }_{{\rm{electrolyte}}}^{(2)}={\chi }_{\rm{cation}}^{(2)}+{\chi }_{\rm{anion}}^{(2)}$, for later convenience. Note that ${{{\chi }}}_{{\rm{electrolyte}}}^{(2)}$ also includes effects due to water displacement, and although the formation of ion pairs has not been explicitly included, it could, in principle, be added as additional contributions to ${{{\chi }}}_{{\rm{electrolyte}}}^{(2)}$ or absorbed into ${{{\chi }}}_{{\rm{anion}}}^{(2)}$.

This interpretation differs from the conventional EDL in which cationic and anionic contributions are entangled. Within the EDL picture, it is assumed that the local electric field created by the charge separation at the interface induces a net average orientation of the O–H transition dipole moments of the water molecules in the topmost layers. However, such a scenario cannot account for the simultaneous decrease and increase in the signal around 3,000 and 3,500 cm^−1^, respectively. Two spectral changes with contrary signs require alignment with opposite orientations, a scenario that cannot be described in the framework of the EDL picture.

**Fig. 3:**
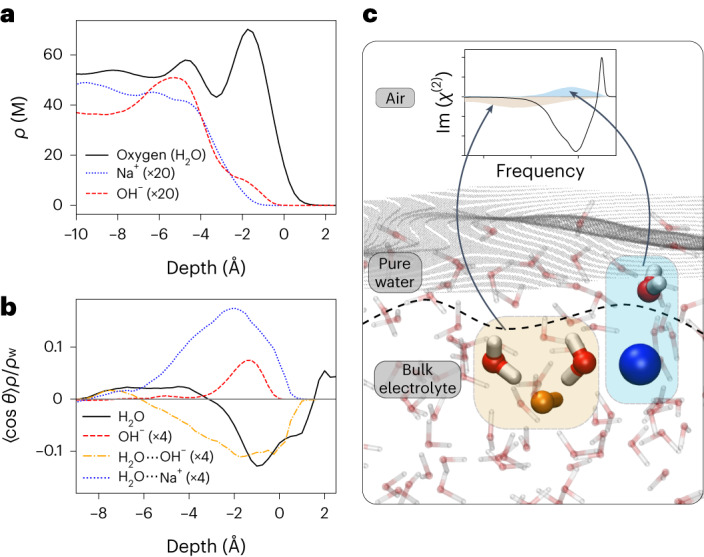
Microscopic analysis of the air/NaOH(aq.) interface. **a**,**b**, Depth profiles of the densities (**a**) and the average angle of the H–O–H bisector of water molecules and OH^−^ bond axis with respect to the surface normal (**b**) for the 2.3 M NaOH solution. The origin point is defined as the position of the instantaneous liquid interface. In **a**, oxygen atoms of the water molecules, Na^+^ and OH^−^ are plotted in black, blue and red, respectively. In **b**, water molecules without ions in their vicinity are in black, OH^−^ in red, water molecules in the vicinity of Na^+^ ions in blue and water molecules in the vicinity of OH^−^ ions in orange. A negative (positive) <cos*θ*> indicates a net down-orientation towards the bulk of the solution (up-orientation towards the air). Values are scaled by the density relative to the water density. Some curves are further scaled for clarity. **c**, A representative snapshot of the MD simulations. Na^+^ and OH^−^ are highlighted in blue and orange, respectively. Selected water molecules coordinated to the ions that are responsible for the observed changes in the HD-VSFG spectrum are also highlighted. The instantaneous air/liquid interface is depicted by a grey surface and the dashed lines represent the interfaces between pure water and bulk electrolyte solution. The H_2_O···OH^−^ and H_2_O···Na^+^ water molecules that give rise to the changes in the VSFG spectra have opposite polarization and lie in an interfacial region depleted of ions. For clarity, H_2_O···OH^−^ and H_2_O···Na^+^ water molecules giving rise to the changes in the VSFG spectra are marked with light-blue and orange areas, respectively. Source data

To further explore the ion distribution near the interfaces, we computed the density profiles for the different species, which are displayed in Fig. 3a. Both Na^+^ and OH^−^ ions are repelled from the interface, giving rise to subsurface enrichment. Figure 3b presents the orientation profile for water, OH^−^ and water molecules coordinated to OH^−^ (H_2_O···OH^−^) and Na^+^ (H_2_O···Na^+^). The water orientation curve shows a slightly negative orientation, in agreement with the literature^42^. The few OH^−^ anions within 3.5 Å of the instantaneous water interface show a clear net orientation with their hydrogen atoms pointing towards the vapour phase. However, the contribution to the overall signal is minor, because only very few OH^−^ groups are present at this depth. Indeed, most OH^−^ anions are over 3.5 Å below the instantaneous water interface, without a net orientation. The orientation of the coordinated water is consistent with the predicted VSFG spectra: H_2_O···OH^−^ molecules point, on average, down to the solution, whereas H_2_O···Na^+^ water molecules are oriented upwards, towards the interface. Importantly, most of the net orientation of the coordinated builds up within a region void of Na^+^ and OH^−^ ions.

With all the previous observations in mind, we propose the following stratification model, as depicted in Fig. 3c (MD snapshot). The lack of ions on the topmost layer creates a thin internal layer of ‘pure’ water. The water molecules inside this thin layer, which are next to ions, show a specific orientation along the surface normal in accordance, causing the observed changes in the Im(*χ*^(2)^) spectra. On the one hand, in the case of Na^+^ ions, due to the spherical symmetry of the cation, only one orientation, in which the oxygen atoms reside closest to the cation, is favoured. On the other hand, and as mentioned earlier, most OH^−^ ions are located 3.5 Å below the instantaneous air/water interface. The hydrogen atom of the OH^−^ motif does not act as hydrogen-bond donor^38^, and the oxygen atom serves as a strong hydrogen-bond acceptor, able to accept between three and five hydrogen bonds per atom^36^. Thus, on average, OH^−^ ions align more water molecules pointing towards the solution than to the interface. Last but not least, inside the bulk region, the molecular orientations become randomized, producing a vanishing net orientation and therefore no additional SFG signal. In Supplementary Section 4 we present a simple one-dimensional model that showcases how the absence of ions in one region of space can lead to a distinct water alignment, as we propose here.

### Generalization of the stratification picture to simple electrolytes

Until this point, we have restricted our focus to the aqueous NaOH solution. If the proposed picture were general and a relevant phenomenon for other electrolytes, it should be possible to find spectroscopic evidence. Thus, to verify our microscopic interpretation, we carried out HD-VSFG measurements for a series of halogen salts with varying strengths of the water–anion interaction.

**Fig. 4:**
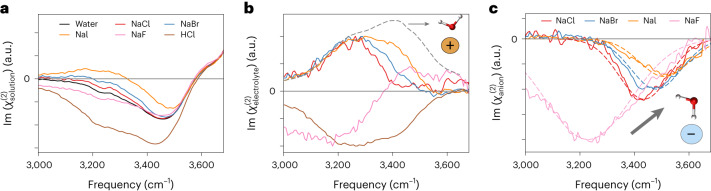
Deconvolution of HD-VSFG into cationic- and anionic-induced spectral contributions. **a**, Imaginary part of the HD-VSFG spectra, ${\rm{Im}}\left({\chi }_{{\rm{solution}}}^{(2)}\right)$, obtained for H_2_O (black), 1 M NaF (pink), 1 M NaCl (red), 1 M NaBr (blue), 1 M NaI (orange) and 1 M HCl (brown) aqueous solutions. **b**, Electrolyte contributions to the second-order response computed from ${\chi }_{{\rm{electrolyte}}}^{(2)}={\chi }_{{\rm{solution}}}^{(2)}-{\chi }_{{\rm{water}}}^{(2)}$. The signals are normalized to the maximum value. The geometric mean of Raman and IR signals for H_2_O, ${({I}_{\rm{IR}}\times {I}_{\rm{Raman}})}^{1/2}$, is depicted by grey dashed line. **c**, Estimated anionic contributions computed from ${\chi }_{{\rm{anion}}}^{(2)}={\chi }_{{\rm{electrolyte}}}^{(2)}-{b}({I}_{\rm{IR}} \times {I}_{\rm{Raman}})^{1/2}$, where *b* is a scaling factor. Gaussian fits to guide the eye are shown as dashed lines. The frequency trend observed for the anionic contributions is consistent with the hydrogen-bond strength expected for the halide series and agrees with the predictions of the stratification picture. Source data

We show the HD-VSFG spectra for HCl, NaCl, NaBr and NaI aqueous interfaces in Fig. 4a. Because the spectral changes induced by the addition of ions are proportional to their concentration (Supplementary Section 1), the total signal can be decomposed into a linear combination of the water, ${\chi }_{{\rm{water}}}^{(2)}$ and ${\chi }_{{\rm{electrolyte}}}^{(2)}$, as shown in equation (1) (that is, ${\chi }_{{\rm{electrolyte}}}^{(2)}={\chi }_{{\rm{solution}}}^{(2)}-{\chi }_{{\rm{water}}}^{(2)}$). The observed linearity with concentration suggests that ion pairs do not form in an appreciable amount. However, we anticipate that a notable departure from the present model at concentrations much greater than 1 M might occur depending on the specific electrolyte’s surface affinity, its tendency to form ion pairs and, ultimately, its solubility. Figure 4b should, in principle, originate from a superposition of a positive signal emerging from the water aligned towards the interface due to its interaction with Na^+^ ions (${\chi }_{{\rm{cation}}}^{(2)}$), with a negative signal arising from the water alignment induced by the anions (${\chi }_{{\rm{anion}}}^{(2)}$). For NaOH, these different contributions are nicely separated, owing to the greater frequency separation between the different water molecules near cations and halide anions.

To test this assumption, we approximate ${\rm{Im}}({\chi }_{{\rm{cation}}}^{(2)})\propto ({I}_{\rm{IR}} \times {I}_{\rm{Raman}})^{1/2}$, where *I*_IR_ and *I*_Raman_ represent the IR and Raman intensities, respectively^13,43^. This approximation is validated by the resemblance between the constructed ${({I}_{\rm{IR}}\times {I}_{\rm{Raman}})}^{1/2}$ spectrum and the spectra shown in Fig. 2c (also Supplementary Fig. 13) and is supported by the following reasoning. In scenarios with a static electric field polarizing water or water molecules interacting with cations, but hydrogen-bonded to other water molecules, the water O–H stretch spectrum is governed by the intermolecular water–water interactions^41^. Thus, the response of water near cations or subject to a static field should resemble the spectral features present in bulk water. This comparison has recently been used successfully to analyse the silica/water interface, demonstrating the validity of such an approximation in situations where a bulk-like response is expected^44^.

The ${\chi }_{{\rm{anion}}}^{(2)}$ spectra contributions, obtained as ${\chi }_{{\rm{anion}}}^{(2)}={\chi }_{{\rm{electrolyte}}}^{(2)}-{\chi }_{{\rm{cation}}}^{(2)}$, are displayed in Fig. 4c. The negative contribution follows the expected trend and is redshifted along the series I^−^ → Br^−^ → Cl^−^ → F^−^, which follows the increase of the relative strength of the corresponding hydrogen bonds^45^. In accordance with the proposed modelling, stronger anion–water interactions give rise to a relatively stronger negative contribution. Similar to what is observed in gas-phase clusters^46,47^, the F^−^ anion forms a remarkably strong hydrogen bond that blueshifts the O–H stretch spectrum by more than 200 cm^−1^ compared to the other halides.

We stress that the EDL interpretation, which attributes the alignment to the local field generated by the ion separations and disregards explicit water–ion interactions, cannot explain the changes observed in the differential spectra for these electrolytes (Fig. 4b).

Figure 4 shows data from the HCl solution. As mentioned earlier, its ${\chi }_{{\rm{electrolyte}}}^{(2)}$ spectrum shows a monotonic change characterized by a decrease of the signal in the hydrogen-bonded O–H stretch region below 3,600 cm^−1^. Together with the fact that the free O–H signal decreases markedly with concentration (Fig. 1g and Supplementary Fig. 12), we believe that, in this system, an EDL is indeed formed, and the field induced by the charge separation actually determines the interfacial structure. Although we suspect that the water coordinated to the Cl^−^ anion should, in principle, create a negative contribution, as it does for the other cases, the strong signal arising from proton-induced alignment masks it entirely.

In Supplementary Information, we also report the data obtained for CsF, MgSO_4_, Na_2_SO_4_, MgCl_2_ and NaClO_4_ electrolytes. In all these cases except for NaClO_4_, the amplitude of the free O–H band remains, within experimental uncertainty, unaffected up to 1 M, showing a lack of ions at the topmost water layer (Supplementary Fig. 12). The determined ${{\rm{\chi }}}_{{\rm{electrolyte}}}^{(2)}$ for CsF shows positive and negative regions, which resemble the NaOH and NaF cases. Although the assumption of the formation of an EDL again fails to rationalize simultaneous spectral changes with different signs, the picture based on the surface stratification provides a straightforward interpretation: the positive and negative peaks arise from water coordinated to the cations and anions, respectively. The deconvolution of the ${{\rm{\chi }}}_{{\rm{anion}}}^{(2)}$ for the F^−^ anion of CsF agrees with the one obtained for NaF (Supplementary Fig. 14) and, in a similar way, the ${{\rm{\chi }}}_{{\rm{anion}}}^{(2)}$ contribution of Cl^−^ obtained from the MgCl_2_ data is in good agreement with that of NaCl (Supplementary Fig. 15). This demonstrates the robustness of the proposed analysis. The results of MgSO_4_ and Na_2_SO_4_ are presented in Supplementary Fig. 16, and, unlike the previous cases, the ${{\rm{\chi }}}_{{\rm{anion}}}^{(2)}$ signal depends on the cation identity. Because the spectral shape reports on the hydrogen-bonding connectivity around the sulfate^48,49^, this result suggests that the observed difference is due to the formation of (solvent-separated) ion pairs in MgSO_4_ (ref. ^50^). The NaClO_4_ solutions show the strongest perturbation of the VSFG signal, and, similar to HCl, a rapid decrease in the free O–H signal with the addition of electrolyte. We therefore conclude that the ClO_4_^−^ is localized on top of the water and, indeed, forms an EDL. Similar behaviour is expected for SCN^−^ and other organic ions^51,52^.

Finally, to verify the emerging picture, we also performed ab initio MD simulations for the sodium halide salts and computed the corresponding density profiles. As shown in Supplementary Fig. 22, the air/liquid interface of the NaF, NaCl and NaBr solutions is depleted of ions, leading to a subsurface enrichment and the aforementioned interfacial stratification. This effect is less pronounced for the NaI solution, which appears to be an intermediate situation between the rest of the halides and the proton. We note that the presented ab initio MD density profiles are largely in agreement with the simulations reported in ref. ^22^, with classical simulations with thermodynamically consistent force fields as reported in ref. ^53^, and with calculations based on continuum models^54,55^. The unperturbed free O–H signal, despite the presence of NaI, reveals that the iodide ion, as opposed to the perchlorate and the hydrated proton, is not very surface-active (Supplementary Fig. 12). Moreover, the spectral changes as a function of NaI concentration are linear (Supplementary Fig. 11), as opposed to the nonlinear response observed for surface-active (ClO_4_^−^ and H^+^) ions. Combined, these observations strongly suggest that the hypothetical local field created by the charge separation according to the EDL picture plays a minimal role. Thus, we analyse NaI in the same way as the other alkali halides (also Supplementary Section V).

In summary, high-level HD-VSFG, in combination with ab initio simulations, in this case, aided by NN, has proven to be an invaluable tool to contribute to the molecular-level understanding of liquid surfaces. Unlike other techniques, such as electron spectroscopy^56^ and second harmonic generation^57^, which provide a relatively coarse-grained view, HD-VSFG has vibrational resolution. The quantitative experimental results reported in this Article for such a large and diverse set of electrolytes, together with the high accuracy of the measurements (Supplementary Fig. 26), enabled the observation of the subtleties of the water–ion interactions, and enabled a critical revision of the domain of applicability of the established EDL picture. More specifically, assuming the formation of an EDL, water molecules within the EDL are predicted to polarize in a single direction—either pointing upwards or downwards. Although it is possible to differentiate the effects of water interacting with ions of varying charges, these contributions would consistently exhibit the same polarity. In contrast, the stratified model accounts for dual polarization, where water can simultaneously orient both upwards and downwards, in alignment with the experimental observations. The stratification picture and the importance of water–ion interactions demonstrated in this Article substantially expand on current textbook descriptions, providing powerful insights towards resolving the air/water interface puzzle and understanding chemical reactivity at this ubiquitous interface^58,59^.

## Methods

### Ab initio simulations

Density functional theory calculations were carried out with the CP2K package^62^ at the revPBE-D3(0) level of theory. This choice has been shown to provide an excellent compromise in terms of the description quality of interfacial water and the required computational cost^63^. We employed the TZV2P basis sets. The core electrons were described by the Goedecker–Teter–Hutter pseudopotential, and the real-space density cutoff was set to 320 Ry. We used a simulation cell with dimensions of 16.63 × 16.63 × 44.10 Å. The 1.1 M, 2.3 M and 4.5 M and 7.5 M NaOH solutions were modelled by a simulation cell containing a slab made of 162, 160, 156 and 152 water molecules and 2, 4, 8 and 12 NaOH molecules, respectively. These NaOH(aq.) slabs correspond to nominal concentrations of 0.675 M, 1.25 M, 2.50 M and 3.75 M, respectively. The slab thickness was found to be ~18 ± 1 Å in all cases. The simulations for NaF, NaCl, NaBr and NaI were performed in larger simulation cells with dimensions of 14.4 × 14.4 × 70.0 Å and were modelled by a slab made of 248 water molecules and six sodium halide molecules. In these cases, the slab thickness was found to be ~38 ± 1 Å. The MD simulations for the sodium halide salts were performed with the CP2K package. For each salt, we prepared ten different initial coordinates and carried out a total of 400 ps in the canonical (NVT) ensemble controlled by a stochastic velocity rescaling thermostat (*τ* = 300 fs) with a target temperature of 300 K to ensure the correct equilibration of the ion distribution.

### NN training

The training of the Behler–Parinello high-dimensional NN was performed using the n2p2 code^64,65^ following the active-learning strategy outlined in ref. ^66^. The reference data were obtained from two independent 20-ps-long microcanonical (NVE) trajectories for each concentration, previously thermalized at 300 K. We trained four different NNs corresponding to pure water, and NaOH(aq.) with nominal concentrations of 1.25 M, 2.50 M and 3.75 M. We also tried to obtain a NN for higher concentrations, namely 5.0 M NaOH, but could not get a stable model that could run for more than 100 ps. The simulations for 0.675 M NaOH(aq.) were performed with the NN trained on 1.25 M NaOH(aq.) data without any stability problem. The training procedure can be summarized as follows. We started the training with 40 randomly picked structures and trained six different NN models to form a committee model^67^. The disagreement among the NN models was used to identify the most relevant configurations, which were added by batches of 20, until the disagreement across could not be improved further. In total, we collected around 300 structures, obtaining energy and force root-mean-square errors for both training and test sets below 1 meV per atom and 100 meV Bohr^−1^, respectively. These overall accuracy estimates are known to yield an accurate representation of the reference potential energy surface^32^. Training curves and force–force correlation plots are presented in Supplementary Information. It is noteworthy to mention that the NN architecture employed in this Article does not include long-range effects explicitly^68,69^. However, because we used a rather small slab and a 6-Å cutoff to describe the atomic environments, which effectively becomes 12 Å in the force calulations^32^, most parts of the electrostatic interactions can be described adequately^70^. Indeed, a similar architecture has been used to model bulk aqueous NaOH solutions and obtained very good agreement with experimental results^36,71,72^.

### NN MD simulations

The production runs for the NaOH solutions were performed with the i-PI program^73^ connected to the LAMMPS package^65,74^. For each concentration, we prepared 40 different initial coordinates and carried out a total of 1 ns in the NVT ensemble controlled by a stochastic velocity rescaling thermostat^75^ (*τ* = 300 fs) with a target temperature of 300 K to ensure the correct equilibration of the ion distribution. The instantaneous interfaces were calculated using the Willard–Chandler method^76^. The ssVVCF^30^ were calculated using a Hahn window of 1 ps and obtained by averaging 40 independent 200-ps trajectories carried out from uncorrelated initial coordinates extracted from the NVT trajectories. The time step employed for the integration of the equation of motions was in all cases 0.5 fs. Unless stated otherwise, the classification as either OH^−^ or water molecule was performed by assuming that each hydrogen was covalently bound exclusively to its nearest oxygen atom^36^. We observed around 100 OH^−^ transfer events per OH^−^ motif in typical 200-ps NVE trajectories (Supplementary Fig. 25). Finally, we considered a water molecule to be coordinated to OH^−^ (H_2_O···OH^−^) or to Na^+^ (H_2_O···Na^+^) if the oxygen–oxygen distance was below 3.0 Å or if the oxygen–sodium distance was below 3.5 Å, respectively. We verified that slightly different cutoff distances did not qualitatively affect the reported results.

### Sample preparation

HCl (36.5%) was obtained from Alfa Aesar. NaOH (>98%), NaF (>99%), NaCl (>99%), NaBr (>99%), NaI (>99%), CsF (>99%), CsCl (>99%,), MgCl_2_ (>99.99%), MgSO_4_(>99.99%), Na_2_SO_4_ (>99.99%) and NaClO_4_ (>99.99%) were obtained from Sigma Aldrich. These chemicals, except NaF, NaCl and NaBr, were used without further purification in the study. NaF, NaCl and NaBr were baked in an oven for 8 h at 650 °C before the experiments to remove organic contaminants. Pure water was obtained from a Milli-Q system (resistance of 18.2 MΩ cm). By dissolving the salt with pure water, we obtained various concentrations of salt solutions. NaI solution was freshly prepared 1 h before measurement to avoid oxidation of iodide ions. Before measurements, all solutions were covered with aluminium foil to prevent photochemical reaction from light. All measurements were performed under nitrogen purge conditions.

### HD SFG spectroscopy

For NaOH aqueous solution samples, we use a non-collinear beam geometry with a Ti:sapphire regenerative amplifier laser system (Spitfire Ace, Spectra-Physics, centred at 800 nm, ~40-fs pulse duration, 5-mJ pulse energy, 1-kHz repetition rate). A part of the output was directed to a grating-cylindrical lens pulse-shaper to produce a narrowband visible pulse (8-μJ pulse energy at the sample position, full-width at half-maximum (FWHM) of ~10 cm^−1^), while the other part was used to generate a broadband IR pulse (3-μJ pulse energy at the sample position, FWHM of ~400 cm^−1^) through an optical parametric amplifier (OPA; Light Conversion TOPAS-C) with a silver gallium disulfide (AgGaS_2_) crystal. The IR and visible beams were first focused onto 200-nm-thick ZnO on a 1-mm-thick CaF_2_ window to generate a local oscillator (LO) signal in a similar manner to ref. ^77^. Three beams were collimated with an off-axis parabolic mirror. A fuse-silica glass plate (thickness, 1.5 mm) was placed in the optical path for the LO signal, allowing phase modulation for the LO signal. Then, visible, LO and IR beams were re-focused by an off-axis parabolic mirror at incident angles of 64°, 61° and 50° at the sample solution interface, respectively. The SFG signal from the sample interfered with the SFG signal from the LO, generating the SFG interferogram. The SFG interferogram was then dispersed into a spectrometer (Shamrock 303i, Andor Technology) and detected by a charge-coupled device camera (Newton, Andor Technology). To monitor the height change of the sample surface due to water evaporation, we used a height displacement sensor (CL-3000, Keyence).

For the other electrolyte solutions (that is, NaF, NaCl, NaBr, NaI, CsF, HCl, MgCl_2_, MgSO_4_, Na_2_SO_4_ and NaClO_4_ aqueous samples), we measured the HD-VSFG spectra on a collinear beam geometry using a Ti:sapphire regenerative amplifier (Spitfire Ace, Spectra-Physics, centred at 800 nm, ~40-fs pulse duration, 5-mJ pulse energy, 1-kHz repetition rate). A part of the output was used to generate a broadband IR pulse in an OPA with an AgGaS_2_ crystal (2.5-μJ pulse energy at the sample position, FWHM of ~350 cm^−1^). The other part of the output was directed through a pulse-shaper consisting of a grating–cylindrical mirror system to generate a narrowband visible pulse with a bandwidth of ~13.5 cm^−1^ (10-μJ pulse energy at the sample position). The IR and visible beam were first focused into a 20-μm-thick *y*-cut quartz plate as the LO. These beams were then collinearly passed through a 2-mm-thick SrTiO_3_ plate for the phase modulation and focused onto the sample surface at angles of incidence of 45°. The SFG signal from the sample interfered with the LO signal, generating the SFG interferogram. The SFG interferogram was dispersed into a spectrometer (Teledyne Princeton Instruments, HRS-300) and detected by a liquid-nitrogen-cooled charge-coupled device camera (Teledyne Princeton Instruments, PyLoN).

The complex spectra of second-order nonlinear susceptibility (*χ*^(3)^) were obtained via Fourier analysis of the SFG interferogram and normalized by a *z*-cut quartz crystal. Unless stated otherwise, all measurements were performed with an *ssp* (denoting *s*-, *s*- and *p*-polarized SFG, visible and IR beams, respectively) polarization combination. For our analysis, we used (*χ*^(2)^)_*ssp*_ data without Fresnel factor correction.

## Online content

Any methods, additional references, Nature Portfolio reporting summaries, source data, extended data, supplementary information, acknowledgements, peer review information; details of author contributions and competing interests; and statements of data and code availability are available at 10.1038/s41557-023-01416-6.

### Supplementary information

Supplementary Information Supplementary Figs. 1–26 and Discussion on data analysis and simulations convergence.

### Source data

Source Data Fig. 1 Data corresponding to all panels of Fig. 1.

Source Data Fig. 2 Data corresponding to all panels of Fig. 2.

Source Data Fig. 3 Data corresponding to panels a and b of Fig. 3.

Source Data Fig. 4 Data corresponding to all panels of Fig. 4.

## Data Availability

The data corresponding to the figures and input files to reproduce the ab initio and NN simulations are available at https://gitlab.com/litman90/electrolytesolutions-si. Reference ab initio trajectories used to train the NN models are publicly available at Zenodo (10.5281/zenodo.10214530). Source data are provided with this paper.

## Code availability

All the codes used in this work are freely available and can be found in the corresponding references provided in Methods.
