# Supplementary material for: Surface stratification determines the interfacial water structure of simple electrolyte solutions
Source: Nat Chem. 2024 Jan 15;16(4):644–50. doi: 10.1038/s41557-023-01416-6 (PMC10997511; doi:10.1038/s41557-023-01416-6)
Supplement: Supplementary file 1 — Supplementary Figs. 1–26 and Discussion on data analysis and simulations convergence. [file 41557_2023_1416_MOESM1_ESM.pdf]

nature chemistry

Article

https://doi.org/10.1038/s41557-023-01416-6

Surface stratification determines the interfacial water structure of simple electrolyte solutions

In the format provided by the authors and unedited

Contents

1 Salt-concentration-dependent sum-frequency generation spectra of electrolyte solutions 2
2 Further analysis on ion contributions to Imχ (2) 10
3 NN validation tests.......... 12
4 Illustration of the interface stratification by a simple 1D model 14
5 Density profiles of sodium halides salts 16
6 On the convergence of the VSFG simulations 19
7 On the time scale of proton transfer events. 20
8 On the phase accuracy of the HD-SFG measurements 21

1 Salt-concentration-dependent sum-frequency generation spectra of electrolyte solutions

To obtain the complex $\chi^{(2)}$ spectra for NaCl, NaBr, NaI, NaF, and HCl in Figure 4 of the main text, we performed heterodyne-detected sum-frequency generation (HD-VSFG) measurement at the air/electrolyte interfaces by varying the concentrations of ions. The $\mathrm{Im}\chi^{(2)}$ data are present in panel (a) of Figs. S1-S10. We observed a negative $\mathrm{Im}\chi^{(2)}$ band between 3000 cm^{-1} and 3600 cm^{-1} for the pure water case. While the addition of some of the electrolytes elevate the $\mathrm{Im}\chi^{(2)}$ signal below 3400cm^{-1}, the addition of other electrolytes enhance the negative contribution in the <3400cm^{-1} region.

We further calculate the electrolyte spectra defined as, $\chi^{(2)}_{\mathrm{electrolyte}} = \chi^{(2)}_{\mathrm{solution}} - \chi^{(2)}_{\mathrm{water}}$, to examine whether the ion-induced spectral changes are constant throughout all concentration regions. Panel (b) of Figs. S1-S10 shows the $\mathrm{Im}\chi^{(2)}_{\mathrm{electrolyte}}$ signals for various ions. The amplitude of $\chi^{(2)}_{\mathrm{electrolyte}}$ increases with increasing salt concentrations. Nevertheless, the spectral shapes remain unchanged, as shown by the normalized spectra presented in panel c of Figs. S1-S10. This manifests that the structure of the interfacial water changes independently of the ion concentrations considered here.

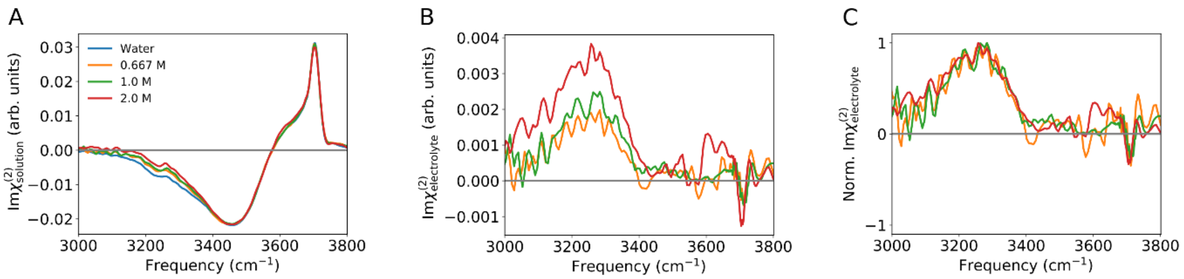

Figure 1. **HD-VSFG spectra for various concentrations of NaCl solution**. (**A**) Imaginary part of $\chi^{(2)}$ spectra, Im $\chi^{(2)}_{\mathrm{solution}}$ (**B**) The difference spectra, Im $\chi^{(2)}_{\mathrm{electrolyte}}$ (**C**) Normalized Im$\chi^{(2)}_{\mathrm{electrolyte}}$ to the same scale for comparison.

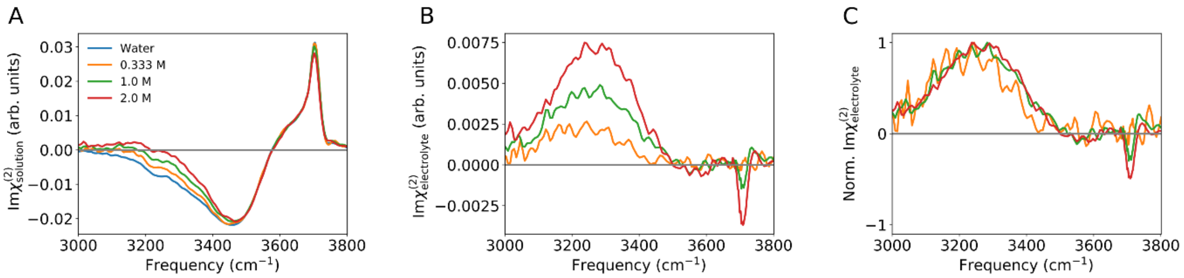

Figure 2**. HD-VSFG spectra for various concentrations of NaBr solution**. (**A**) Imaginary part of $\chi^{(2)}$ spectra, Im $\chi^{(2)}_{\mathrm{solution}}$ (**B**) The difference spectra, Im $\chi^{(2)}_{\mathrm{electrolyte}}$ (**C**) Normalized Im$\chi^{(2)}_{\mathrm{electrolyte}}$ to the same scale for comparison.

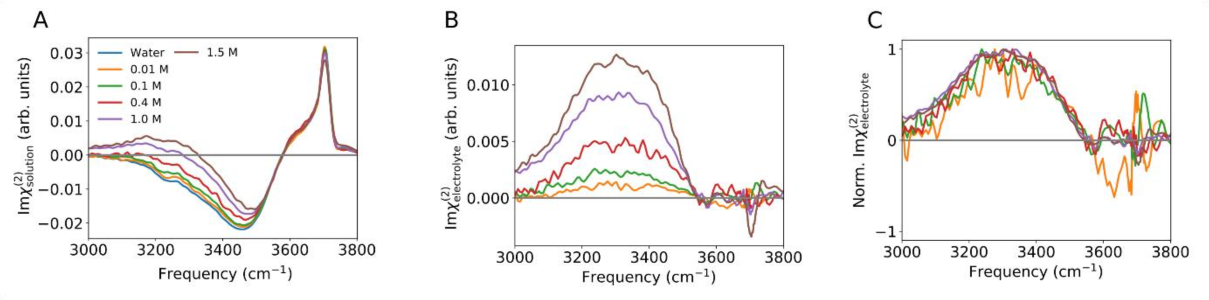

Figure 3. **HD-VSFG spectra for various concentrations of NaI solution.** (**A**) Imaginary part of $\chi^{(2)}$ spectra, Im $\chi^{(2)}_{\mathrm{solution}}$ (**B**) The difference spectra, Im $\chi^{(2)}_{\mathrm{electrolyte}}$ (**C**) Normalized Im$\chi^{(2)}_{\mathrm{electrolyte}}$ to the same scale for comparison.

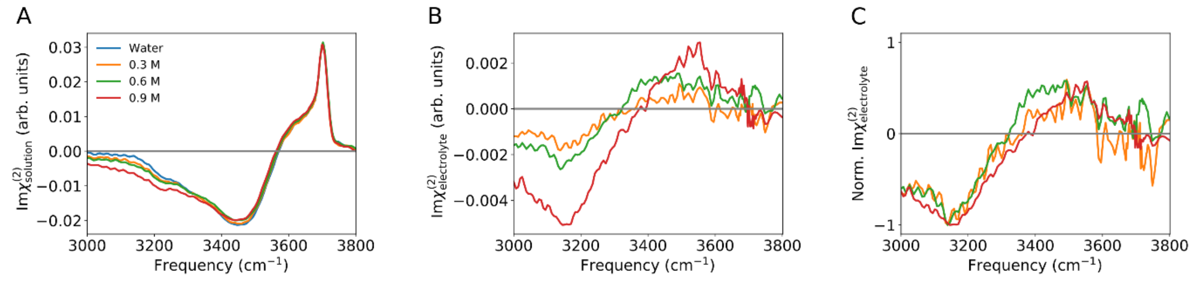

Figure 4. **HD-VSFG spectra for various concentrations of NaF solution** (**A**) Imaginary part of $\chi^{(2)}$ spectra, Im $\chi^{(2)}_{\mathrm{solution}}$ (**B**) The difference spectra, Im $\chi^{(2)}_{\mathrm{electrolyte}}$ (**C**) Normalized Im$\chi^{(2)}_{\mathrm{electrolyte}}$ to the same scale for comparison.

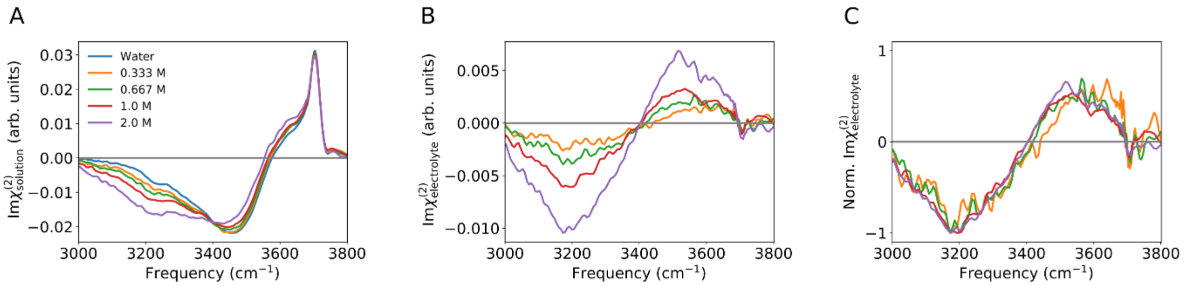

Figure 5. **HD-VSFG spectra for various concentrations of CsF solution** (**A**) Imaginary part of $\chi^{(2)}$ spectra, Im $\chi^{(2)}_{\mathrm{solution}}$ (**B**) The difference spectra, Im $\chi^{(2)}_{\mathrm{electrolyte}}$ (**C**) Normalized Im$\chi^{(2)}_{\mathrm{electrolyte}}$ to the same scale for comparison.

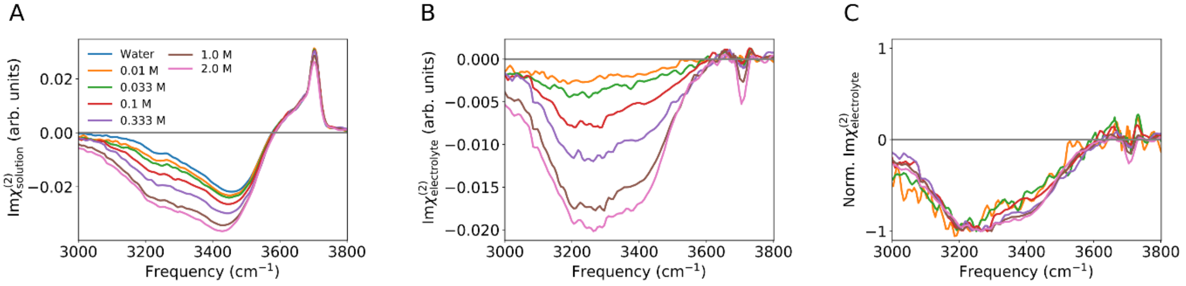

Figure 6. **HD-VSFG spectra for various concentrations of HCl solution** (**A**) Imaginary part of $\chi^{(2)}$ spectra, Im $\chi^{(2)}_{\mathrm{solution}}$ (**B**) The difference spectra, Im $\chi^{(2)}_{\mathrm{electrolyte}}$ (**C**) Normalized Im$\chi^{(2)}_{\mathrm{electrolyte}}$ to the same scale for comparison.

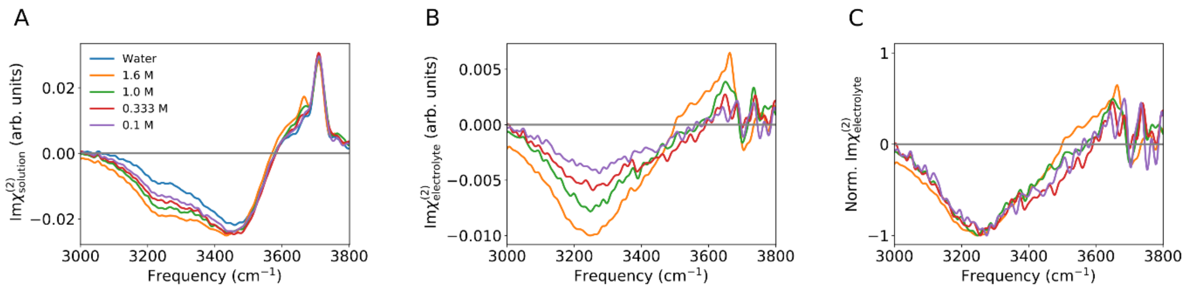

Figure 7. **HD-VSFG spectra for various concentrations of Na_2SO_4 solution** (**A**) Imaginary part of $\chi^{(2)}$ spectra, Im $\chi^{(2)}_{\mathrm{solution}}$ (**B**) The difference spectra, Im $\chi^{(2)}_{\mathrm{electrolyte}}$ (**C**) Normalized Im$\chi^{(2)}_{\mathrm{electrolyte}}$ to the same scale for comparison.

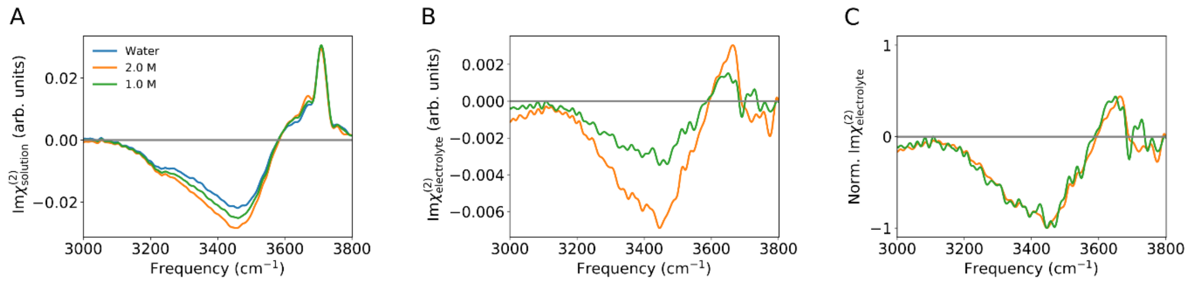

Figure 8. **HD-VSFG spectra for various concentrations of $MgSO_4$ solution** (**A**) Imaginary part of $\chi^{(2)}$ spectra, Im $\chi^{(2)}_{\mathrm{solution}}$ (**B**) The difference spectra, Im $\chi^{(2)}_{\mathrm{electrolyte}}$ (**C**) Normalized Im$\chi^{(2)}_{\mathrm{electrolyte}}$ to the same scale for comparison.

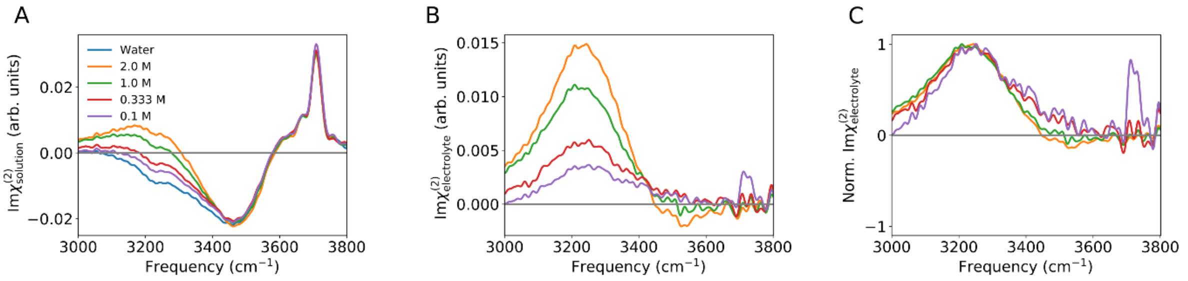

Figure 9. **HD-VSFG spectra for various concentrations of $MgCl_2$ solution** (**A**) Imaginary part of $\chi^{(2)}$ spectra, Im $\chi^{(2)}_{\mathrm{solution}}$ (**B**) The difference spectra, Im $\chi^{(2)}_{\mathrm{electrolyte}}$ (**C**) Normalized $\mathrm{Im}\chi^{(2)}_{\mathrm{electrolyte}}$ to the same scale for comparison.

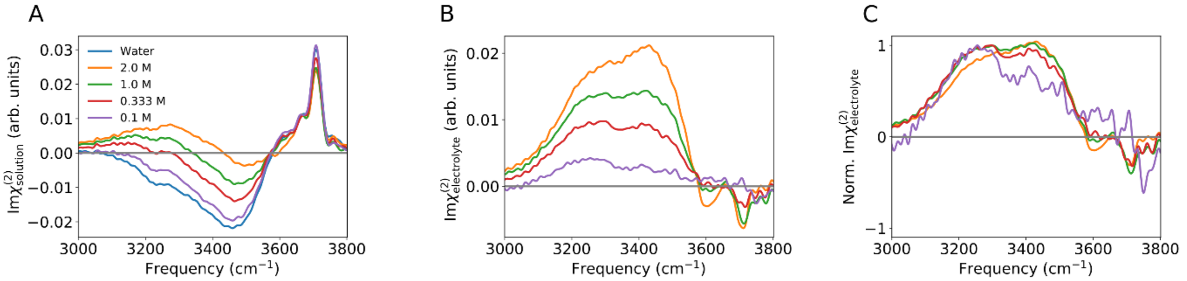

Figure 10. **HD-VSFG spectra for various concentrations of $NaClO_4$ solution** (**A**) Imaginary part of $\chi^{(2)}$ spectra, Im $\chi^{(2)}_{\mathrm{solution}}$ (**B**) The difference spectra, Im $\chi^{(2)}_{\mathrm{electrolyte}}$ (**C**) Normalized $\mathrm{Im}\chi^{(2)}_{\mathrm{electrolyte}}$ to the same scale for comparison.

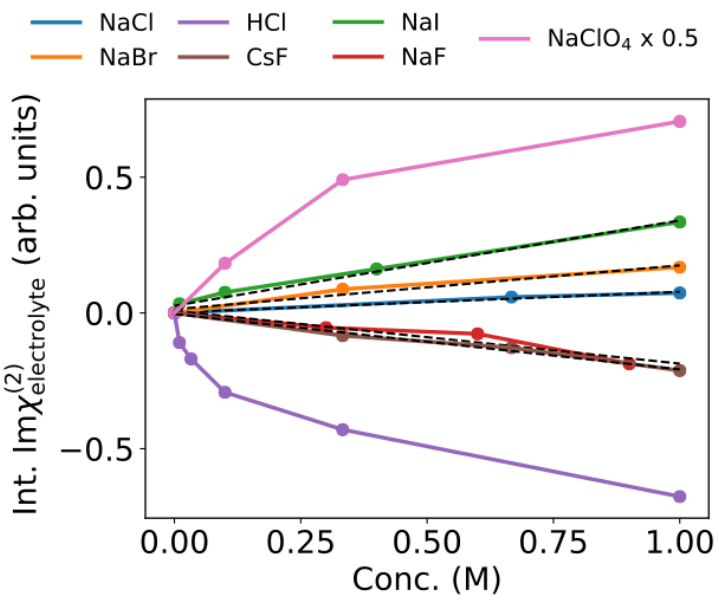

Figure 11. **Concentration dependence of Im $\chi^{(2)}_{\text{electrolyte}}$.** Concentration dependence of integrated Im$\chi^{(2)}_{\text{electrolyte}}$ signals for sodium halide salts, HCl, $HClO_4$ and CsF. Linear fits are depicted by dashed black lines. In all the cases the signal is integrated from 3000 to 3350 cm^{-1}.

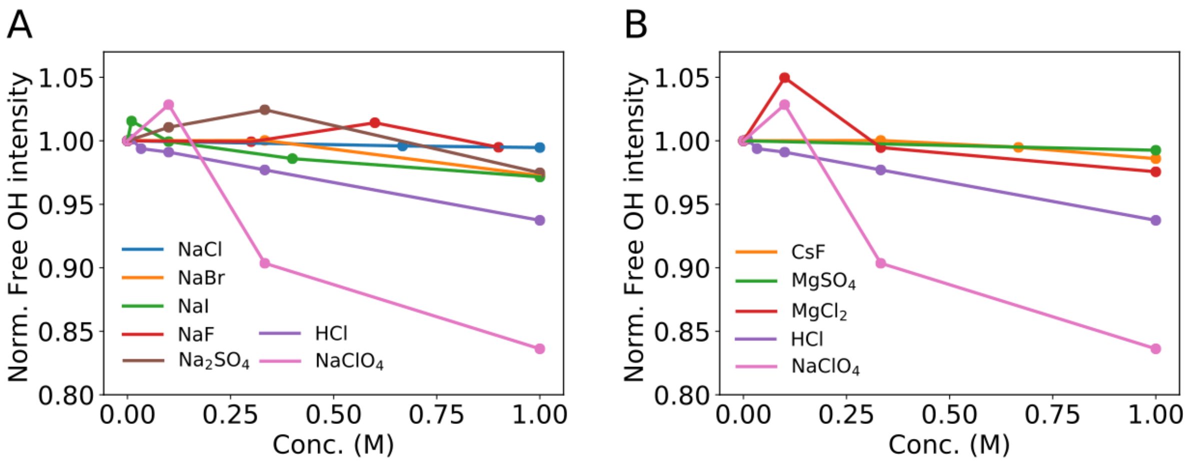

Figure 12. **Concentration dependence of free O-H peak.** Concentration dependence of integrated $\text{Im}\chi^{(2)}_{\text{solution}}$ signals for the different salts. The data is split into two plots showing the HCl and $HClO_4$ curves in both panels to ease visualization. In all the cases, the signal is integrated from 3685 to 3715 cm^{-1}.

2 Further analysis on ion contributions to $\mathrm{Im}\chi^{(2)}$

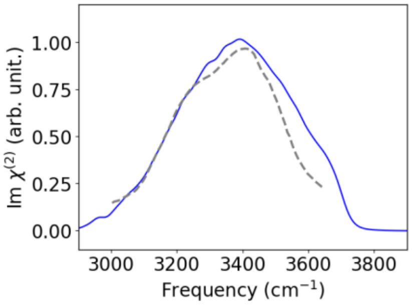

Figure 13. **Comparison of Experimental $(I_{IR} * I_{Raman})^{1/2}$ with Simulated Na⁺-induced Spectral Contribution**. Contribution to the simulated $\mathrm{Im}\chi_c^{(2)}$ spectra by the water molecules in the vicinity of Na^+ ions for NaOH (blue) and the geometric mean of Raman and IR signals of pure water (gray dashed). The former is displaced by -50 cm^{-1}.

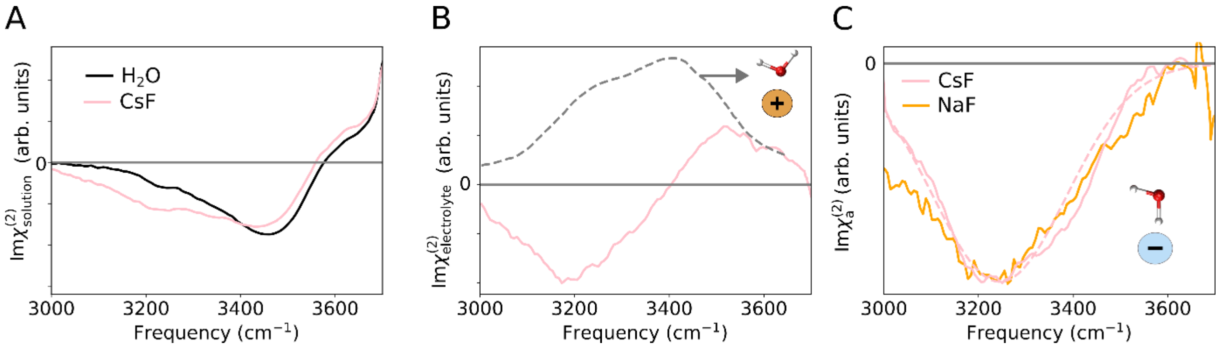

Figure 14. **Deconvolution of HD-VSFG into Cs^+ and F^- induced Spectral Contributions (A)** Imaginary part of VSFG spectra obtained for H_2O (black) and NaF (pink) aqueous solutions. **(B)** Electrolyte contribution computed as $\chi^{(2)}_{\mathrm{electrolyte}} = \chi^{(2)}_{\mathrm{solution}} - \chi^{(2)}_{\mathrm{water}}$. The geometric mean of Raman and IR signals for H_2O, $(I_{IR} * I_{Raman})^{1/2}$, is depicted by grey dashed line. **(C)** Estimated anionic contribution computed as $\chi^{(2)}_{\mathrm{a}} = \chi^{(2)}_{\mathrm{electrolyte}} - b(I_{IR} * I_{Raman})^{1/2}$, where b is a scaling factor. A Gaussian fit to guide the eye are shown as dashed line. $\chi^{(2)}_{\mathrm{a}}$ obtained for the F^- contribution in NaF is shown in orange.

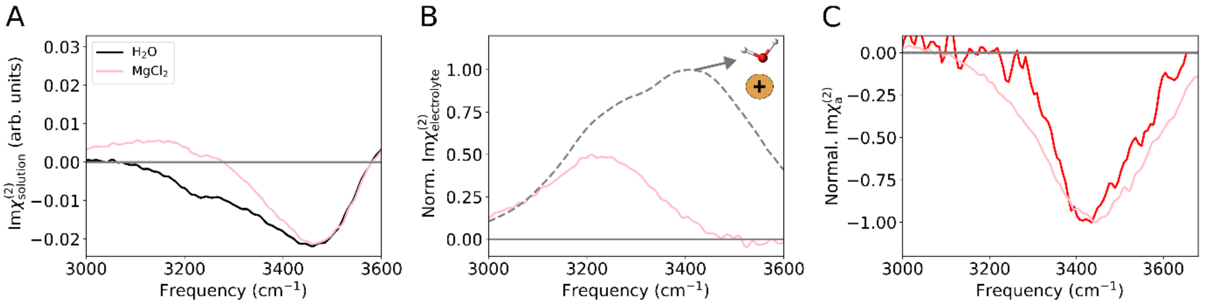

Figure 15. **Deconvolution of HD-VSFG into Mg^{2+} and Cl^- induced Spectral Contributions.** (A) Imaginary part of VSFG spectra obtained for H_2O (black) and $MgCl_2$ (pink) aqueous solutions. (B) Electrolyte contribution computed as $\chi^{(2)}_{\text{electrolyte}} = \chi^{(2)}_{\text{solution}} - \chi^{(2)}_{\text{water}}$. The geometric mean of Raman and IR signals for H2O, $(I_{IR} * I_{Raman})^{1/2}$, is depicted by grey dashed line. (C) Estimated anionic contribution computed as $\chi^{(2)}_{\text{a}} = \chi^{(2)}_{\text{electrolyte}} - b(I_{IR} * I_{Raman})^{1/2}$, where b is a scaling factor. $\chi^{(2)}_{\text{a}}$ obtained for the Cl^- contribution in NaCl is shown in red.

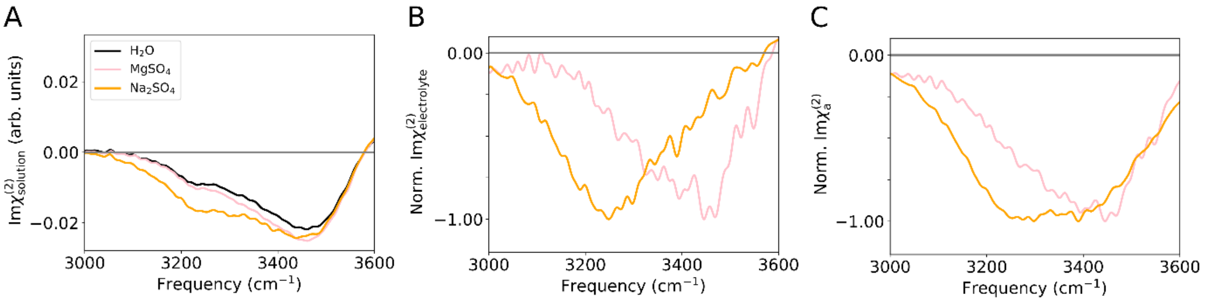

Figure 16. **Deconvolution of HD-VSFG into ionic induced Spectral Contributions**. Same as Figure 14 for $MgSO_4$ (orange) and Na_2SO_4 (pink). The lack of agreement between the spectra in panel (C) indicates that for $MgSO_4$, cationic and anionic contributions to the surface spectrum are not simply additive, consistent with cooperative effects in ion hydration (Tielrooij et al. Science **328**, 1006 (2010)) due to, e.g., (interfacial) ion pairing.

3 Neural network validation tests

Besides the global error obtained on the predicted energies and forces reported in the Methods section, we report in Figs. S17 and S18 the training curves and force-force correlation plots, respectively. We also verified that quality of the predicted vibrational density of states (VDOS). In Figure 19, we compare the predicted VDOS by the NN and the reference data. Even though the reference curves present a relatively large noise due to the small simulation length, a very good agreement between the NN and *ab initio* simulations is obtained. In Figure 20 we compare the predicted VSFG spectra for the pure air/water interface with one previously reported using the same exchange correlation functional.

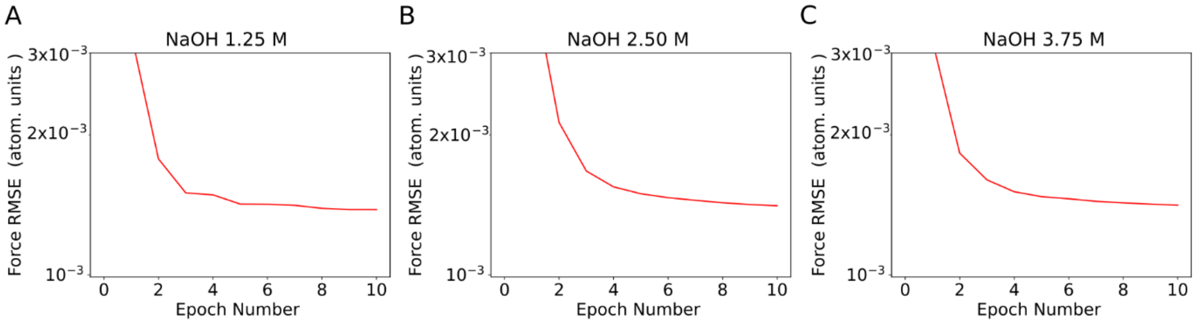

Figure 17. **Training curves of NaOH NN.** Root-mean-square error (RMSE) of total force obtained for the test set as function of epoch during the NN training on the final data set.

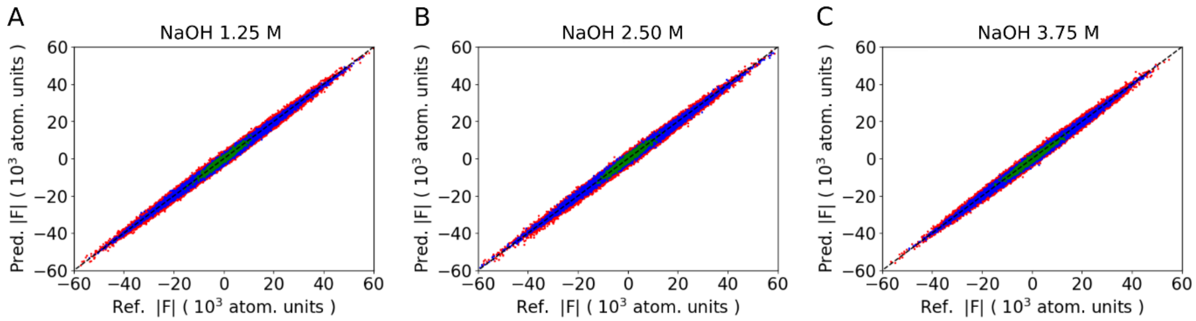

Figure 18. **Force-Force Correlation Plots for NaOH NN.** Atomic force-force correlation plots for each a) NaOH 1.25 M NN, b) NaOH 2.50 M NN, and c) NaOH 3.75 M NN. Atomic forces of

oxygen, sodium and hydrogen atoms are represented by red, green and blue symbols, respectively. Perfect correlation is depicted by a black dashed line in each plot to guide the eye.

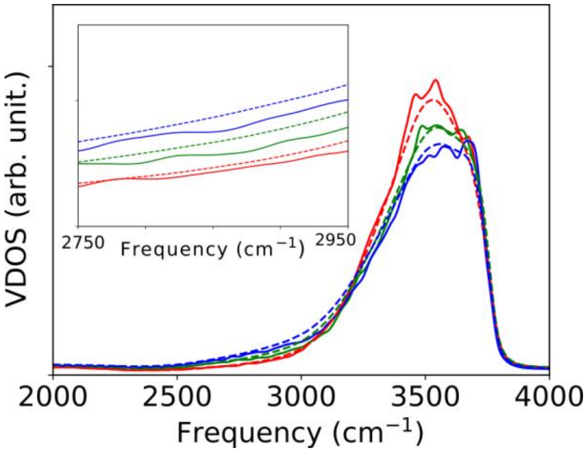

Figure 19. **Vibrational density of states (VDOS) of NaOH(aq) slab structures.** VDOS of NaOH aqueous solutions obtained with the reference *ab initio* trajectories (solid lines) and the NN (dashed lines). NaOH 1.25 M, NaOH 2.50 M, and NaOH 3.75 M are depicted with red, green, and blue colors, respectively.

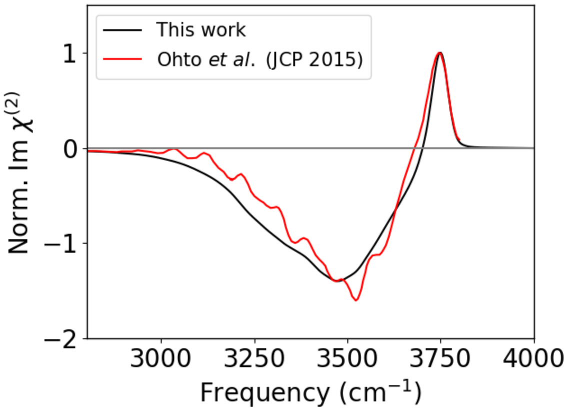

Figure 20. **Validation of NN results for the water/air interface.** Comparison of the predicted VSFG signal of the water/air interface obtained in this work (black line) and the one reported in Ohto et al. *J. Chem. Phys.* 143, 124702 (2015) (red line).

4 Illustration of the interface stratification by a simple 1D model

In this section we describe a simple 1D model to represent the geometrical effects caused by the stratification of the water interface. We consider a 1D slab of length **L** with an inner bulk-like region of length **B**. The bulk region contains water molecules, cations and anions. Importantly, while the ions are constrained between **-B/2** and **B/2**, the water molecules can be found outside of this region. We assume that each ion is solvated by two water molecules at each side and that water-ion distance is ***d***. The numerical simulation goes as follows:

1) Pick a random position between **-B/2** and **B/2** to place the ion and name it **X**
2) Add +1/-1 at a distance **X**+/-**d**. This represents the orientation and position of the water molecules (Note the water molecules can fall outside the [**-B/2,B/2**] region)
3) Repeat steps 1 and 2 until convergence of the corresponding histogram.

In Figure 21a and Figure 21b, we present two cartoons describing one step of numerical modelling for an anion and a cation, respectively. In Figure 21c, we show the results after 20000 iterations. First, the counts in the **[-D/2+d,+D/2+d]** regions averages to zero implying that the lack of net orientation of water molecules inside the bulk-like region. Second, looking at the position **D/2**, positive and negative net orientation are built for the water coordinating cation and anion, respectively. Due to symmetry, the opposite effect is observed at the position **-D/2**. However, in macroscopic samples, where **D** is several micrometres, experiments can easily discriminate both interfaces. Finally, since the vibrational frequencies of water molecules coordinated to anions and cations are normally different, the positive and negative signals emerging from the different 'types' of water molecules do not cancel out and generate a characteristic signature in a real spectrum.

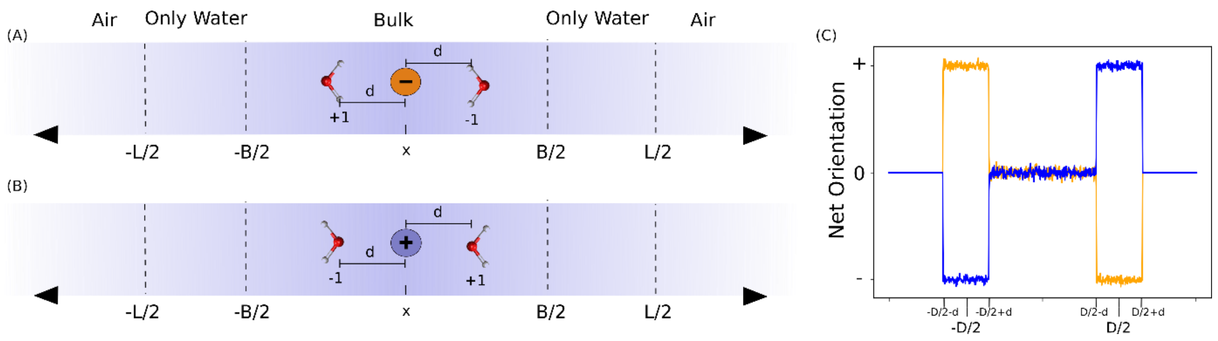

Figure 21. **Description of the 1D model illustration of the stratification at the water/air interface. (A)** Cartoon representing one step of the numerical modelling for an anion **(B)** Same as a for a cation **(C)** Result of the numerical model after 20000 iterations. Anions and cations curves are represented by blue and orange lines, respectively.

5 Density profiles of sodium halides salts

In Figure 22, we present the density profiles obtained from *ab initio* MD simulations for NaF, NaCl, NaBr, and NaI. To further investigate the NaI case, we performed classical force field MD simulations varying the atomic charge of the iodide atom to control its surface propensity. In Figure 23, we present the density and orientation profiles using different values of iodide charges. The idea of using scaled charges is to compensate for the small dielectric constant predicted by the water model and recover the Debye–Hückel law. The 0.85e value is specific to the TIP4P/2005 water model. Vega and co-workers showed that this value of the atomic charge delivers densities, viscosity, diffusion, etc, in better agreement with experiments (Blazques, et al. J. Chem. Phys. **156**, 044505 (2022)). However, the -0.85e is derived in bulk, and since any water model trivially reproduces the vacuum dielectric constant, one would expect at the interface a value closer to the nominal charge (i.e., 1.0). While the simulations with $q(I^-)$=-0.85 and $q(I^-)$=-0.90 show an enhanced I^- concentration at the interface (panels C and D) and single water polarization (panels G and H), the simulations with $q(I^-)$=-0.95 and $q(I^-)$=-1.00 show dual polarization, i.e., simultaneous up- and downward-oriented interfacial water. In all the cases, there is a subsurface enrichment of the cation. By comparing Figure 22 and 23, we find that the classical simulation with $q(I^-)$=-0.95 shows the best agreement with the reference *ab initio* results and conclude that the NaI follows the stratification picture and induces dual polarization.

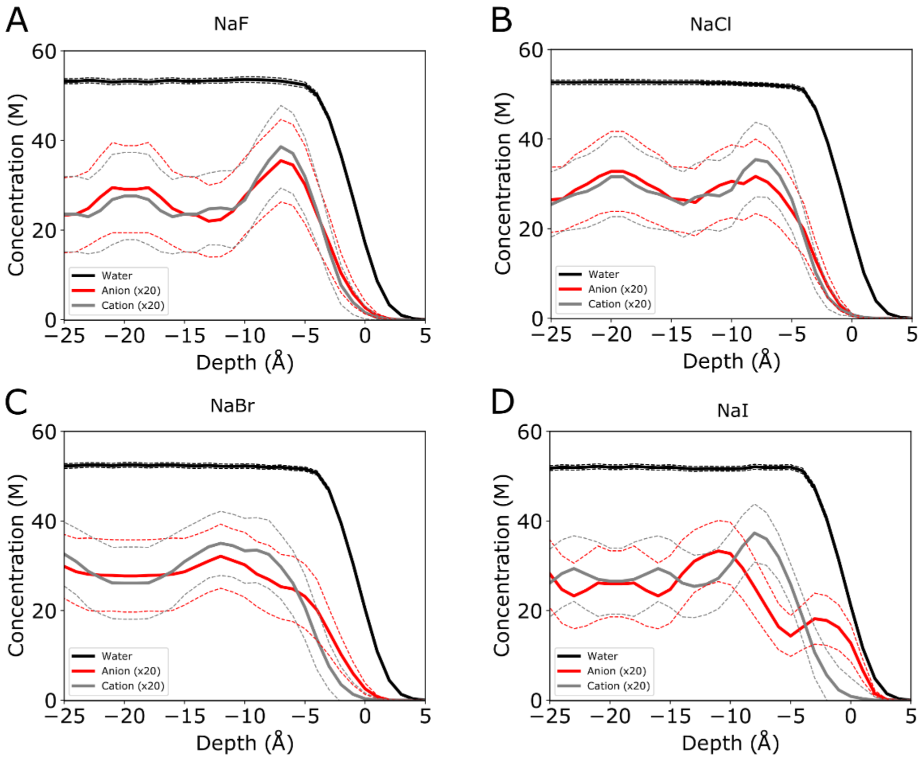

Figure 22. **Average density profiles.** Average density profiles of **(A)** NaF, **(B)** NaCl, **(C)** NaBr, and **(D)** NaI obtained from *ab initio* MD simulations. Mean values and 95% confidence intervals are presented by solid and dashed lines, respectively.

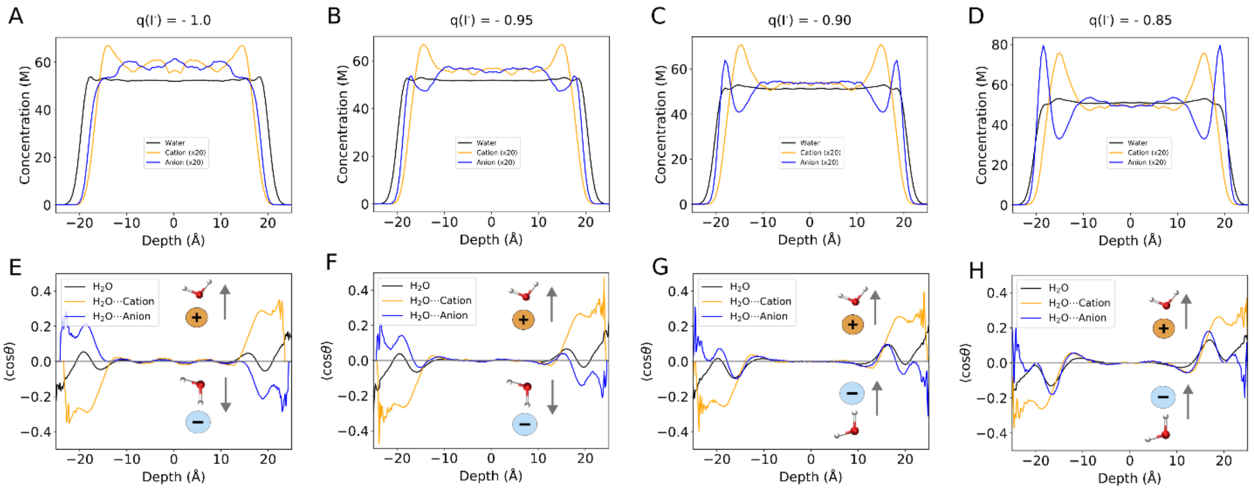

Figure 23. **Average density and water orientation profiles of NaI**. Average density profiles of NaI 2 M obtained from classical molecular dynamics simulations. The force field parameters were obtained from Vega and co-workers (Blazques, et al. J. Chem. Phys. **156**, 044505 (2022)). The charge on the iodide atom was artificially varied from its reference value of -0.85 up to a value of -1.0. **(A,E)** q(I-)=-1.0 **(B,F)** q(I-)=-0.95 **(C,G)** q(I-)=-0.90, and **(D,H)** q(I-)=-0.85 (value reported by Vega and co-workers). Averages were obtained from 160 ns long simulations. Each simulation box contained 512 water molecules, 20 cations and 20 anions. Gray arrows in panels E-H denote the direction of the water molecular dipole for water next to cations and anions. The simulation with q(I$^-$)=-0.95 shows the best agreement with the reference *ab initio* results.

6 On the convergence of the VSFG simulations

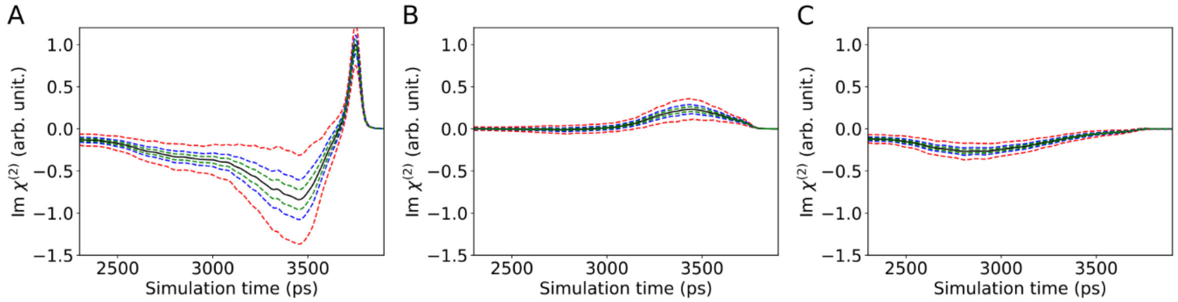

Figure 24. **Convergence of VSFG simulations. (A)** Theoretical prediction of the VSFG spectrum of NaOH 1.25 M based on the ssVVCF methodology. **(B)** Contribution to the theoretical spectra by the water molecules in the vicinity of Na^+ ions **(C)** Contribution to the theoretical spectra by the OH bonds coordinating OH^- anions. In all cases, the black curve represents the mean value for a total of 8.0 ns simulation trajectories. 95% confidence intervals computed for 8.0 ns, 2.0 ns and 0.4 ns are depicted by green, blue, and red dashed lines, respectively.

7 On the time scale of proton transfer events.

We analysed whether the reactive nature of the NN, i.e. its ability to allow bond breaking and formation, was indeed required for the considered systems. In Figure 25, we show the time trace of proton transfer events for one 200ps NVE trajectory. There are around 100 OH^- transfer events per OH^- motif which is consistent with previously reported OH^- lifetimes [Hellström and Behler, *J. Phys. Chem. Lett.* **7**, 3302 (2016)].

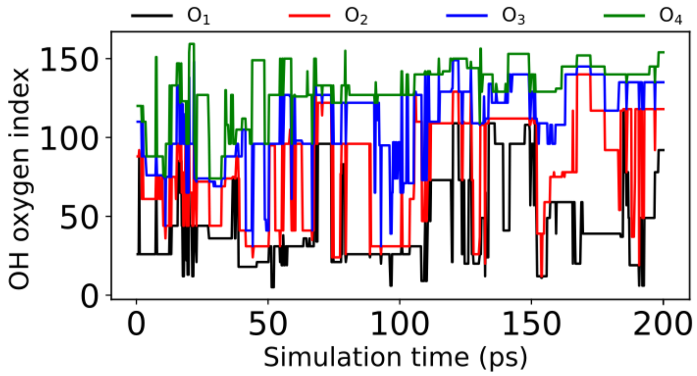

Figure 25. **Time Trace of Proton Transfer Events.** Time evolution of the indexes of oxygen atoms label as OH^- for one 200ps NVE trajectory of NaOH 1.25M. The simulation box contains 4 OH^-, labeled as O_i with i=1-4. The classification of each oxygen atom as either OH^- or water molecule was performed by assuming that each H is covalently bound exclusively to its nearest oxygen atom.

8 On the phase accuracy of the HD-SFG measurements

In Figure 26, we compare the error obtained in our measurements with the predicted one assuming a 10 degrees error in the determination of the phase.

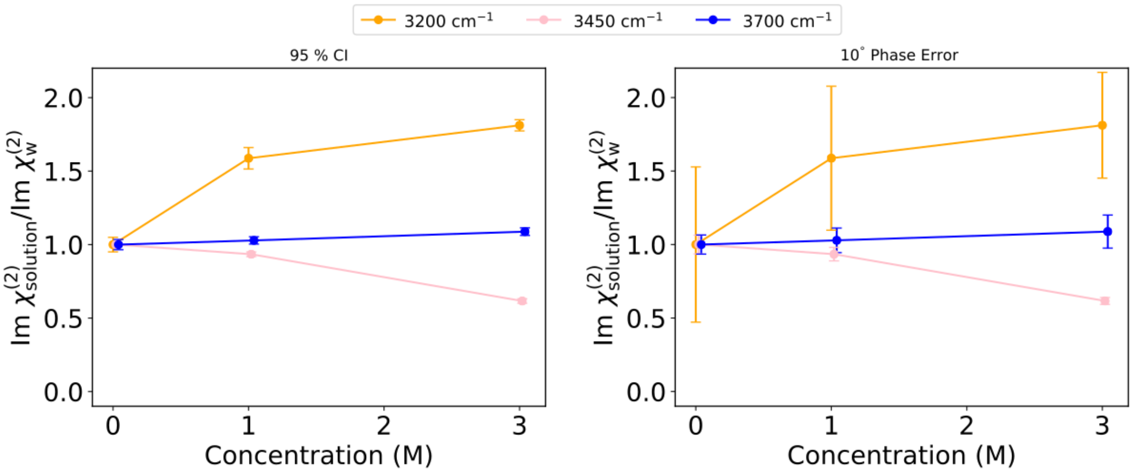

Figure 26. **Dependence of the NaOH Experimental Results on the Phase Accuracy.** Ratio between NaOH aqueous solutions and pure water signal at 3200 cm^{-1} (orange), 3450 cm^{-1} (pink), and 3700 cm^{-1} (blue). Data is presented as mean values over 9 independent data sets. Left: Error bars represent the corresponding 95% confidence interval assuming a two-sided Students' distribution. Right: Error estimated assuming a 10-degrees error in the phase determination.
